# Biofilm-Forming Bacteria Implicated in Complex Otitis Media in Children in the Post-Heptavalent Pneumococcal Conjugate Vaccine (PCV7) Era

**DOI:** 10.3390/microorganisms11030545

**Published:** 2023-02-21

**Authors:** Anastasios Ioannidis, Stylianos Chatzipanagiotou, Niki Vassilaki, Polyvios Giannakopoulos, Despina Hatzaki, Maria Magana, Athanasios Sachlas, George Mpekoulis, Alexandros Radiotis, Michail Tsakanikos, Georgina Tzanakaki, Evangelia Lebessi, Maria N. Tsolia

**Affiliations:** 1 Department of Nursing, Faculty of Health Sciences, University of Peloponnese, 22100 Tripoli, Greece; 2 Department of Clinical Microbiology and Medical Biopathology, Aeginition Hospital, Medical School, National and Kapodistrian University of Athens, 11527 Athens, Greece; 3 Laboratory of Molecular Virology, Hellenic Pasteur Institute, 11521 Athens, Greece; 4 ENT Department, “P. and A. Kyriakou” Children’s Hospital, 11527 Athens, Greece; 5 Department of Microbiology, “P. and A. Kyriakou” Children’s Hospital, 11527 Athens, Greece; 6 Department of Computer Science and Biomedical Informatics, University of Thessaly, 38221 Volos, Greece; 7 National Meningitis Refrence Laboratory, School of Public Health, University of West Attica, 12243 Athens, Greece; 8 Second Department of Pediatrics, School of Medicine, National and Kapodistrian University of Athens, “P. and A. Kyriakou” Children’s Hospital, 11527 Athens, Greece

**Keywords:** otitis media, effusion, children, biofilm, clinical isolates, PCV7, PCV13, vaccination, multiplex PCR, re-operation

## Abstract

**Background**: Chronic media with effusion (COME) and recurrent acute otitis media (RAOM) are closely related clinical entities that affect childhood. The aims of the study were to investigate the microbiological profile of otitis-prone children in the post-PCV7 era and, to examine the biofilm-forming ability in association with clinical history and outcome during a two-year post-operative follow-up. **Methods**: In this prospective study, pathogens from patients with COME and RAOM were isolated and studied in vitro for their biofilm-forming ability. The minimum inhibitory concentrations (MIC) of both the planktonic and the sessile forms were compared. The outcome of the therapeutic method used in each case and patient history were correlated with the pathogens and their ability to form biofilms. **Results**: *Haemophilus influenzae* was the leading pathogen (35% in COME and 40% in RAOM), and *Streptococcus pneumoniae* ranked second (12% in COME and 24% in RAOM). Polymicrobial infections were identified in 5% of COME and 19% of RAOM cases. Of the isolated otopathogens, 94% were positive for biofilm formation. **Conclusions**: This is the first Greek research studying biofilm formation in complex otitis media-prone children population in the post-PCV7 era. High rates of polymicrobial infections, along with treatment failure in biofilms, may explain the lack of antimicrobial efficacy in otitis-prone children.

## 1. Introduction

Otitis media (OM) or middle ear inflammation is a spectrum of diseases, including acute otitis media (AOM), otitis media with effusion (OME), and chronic suppurative otitis media (CSME). OM is one of the most common diseases in young children worldwide. Although OM may resolve spontaneously without complications, it can be associated with hearing loss and life-long sequelae [1]. Acute otitis media (AOM), where the middle ear infection occurs abruptly, causing swelling, redness, and pain, is the second most common pediatric diagnosis in the emergency department after upper respiratory infections [2]. Otitis media with effusion (OME) is conventionally defined as the presence of fluid in the middle ear without signs and symptoms of AOM and is the most common reason for conductive hearing loss in children [3,4]. In the majority of OME cases, effusion is drained through the Eustachian tube (ET) within 3 months; otherwise, in chronic OME (COME, lasting >3 months), myringotomy with or without ventilation tubes (VT) insertion is considered [5]. Recurrent acute otitis media (RAOM) refers to more than three episodes of AOM in a six-month period without residual fluid in the middle ear among the episodes [4]. Children suffering from these two entities are referred to as otitis-prone children. Common therapeutic approaches include the administration of antibiotics or ear surgery [6,7,8]. Since adenoids are considered to be a reservoir of bacteria, being a risk factor for otitis media development, adenoidectomy is always performed [6,9,10]. Bacteria that usually colonize the nasopharynx may turn pathogenic following viral upper respiratory tract infections (URTIs). Common AOM pathogens, including *Streptococcus pneumoniae*, non-typeable *Haemophilus influenzae*, and *Moraxella catarrhalis*, are most often isolated from nasopharyngeal, adenoidal, or middle-ear fluid specimens [11,12]; however, the COME fluid is usually perceived as aseptic due to the presence of “unculturable” or cultivation-demanding (fastidious) bacteria, the intracellularly-hosted microorganisms (viruses or bacteria), and the biofilm-forming bacteria that give negative culture results [4,13,14,15,16,17].

In nature, bacteria tend to exist in complex yet well-organized, surface-attached communities known as biofilms. In the biofilm state, bacteria express different genes compared to their planktonic counterparts and develop self-defending properties against the host immune system, mechanical shear forces, radiation, heat, and antimicrobials [8,18]. In particular, bacteria located in the core of the biofilm remain metabolically inactive (persister cells) and protected from environmental threats by the outer layers, thus preserving the potential of shedding and infecting new niches [8].

*S. pneumoniae* capsular polysaccharide (CPS) is reported to predispose colonization and biofilm formation affecting the nasopharyngeal and middle ear microbiota [19]. It is already reported that pneumolysin and other inflammatory mediators, which are products of pneumococcal infection, cause local mucosal damage through biochemical and structural changes in these tissues [20]. Moreover, the presence of *S. pneumoniae* in the middle ear of animals led to serious damage in both inner and outer hair cells [20]. The above data suggest that the pathogen-derived damage in these tissues seems to be responsible for increased susceptibility to future complex otitis caused by non-typeable *H. influenzae* and other AOM pathogens [10,21].

Polymerase chain reaction (PCR) identifies bacterial DNA in as high as 80% of the middle ear fluid of OME patients even four weeks after the completion of antimicrobial therapy. The detected amplifiable DNA is derived from viable bacteria, as suggested by animal model experiments showing that purified bacterial DNA or DNA from pasteurized bacteria, co-inoculated with infectious organisms in the middle ear, does not persist for more than 2 days [22]. Moreover, combinations of extended culture-confocal microscopy techniques have demonstrated the presence of viable bacteria in more than 90% of effusion fluid specimens [4,17]. Combined, the above data indicate the importance of bacteria and biofilms in the pathogenesis of OME condition [10,23,24,25].

The introduction of immunization with the pneumococcal conjugate vaccines has had a considerable impact on the epidemiology of pneumococcal infections in the pediatric population [26,27,28]. The reduction of the pneumococcal burden aims to alleviate the sequelae from this major pathogen [29]. Moreover, there is evidence of protection against otitis-prone children, and it is documented that the impact is not only against vaccine serotypes but has a wider cumulative impact on OM [29] even when it is caused by non-vaccine serotypes and *H. influenzae* [20].

In the current study, we investigated the microbiological profile of otitis-prone children in the post-PCV7 era with the use of conventional and molecular methods. We also examined the ability of isolated pathogens to form biofilms in vitro. The results were correlated with the patient’s clinical history as well as the outcome of a two-year post-operative follow-up.

## 2. Materials and Methods

### 2.1. Study Population

A four-year prospective study was conducted among children admitted to the Ear-Nose-Throat department of Athens General Children’s Hospital “P. & A. Kyriakou” for ventilation tube (VT) insertion along with adenoidectomy either with chronic OME (COME, *n* = 236), or RAOM (*n* = 37). This hospital serves a population of >500,000 children younger than 14 years of age. The study was approved by the hospital’s Research Ethics Committee (No 9392/10.06.11), and written informed consent was sought from the parents. All procedures were in accordance with the ethical standards of the Helsinki Declaration of 1975, as revised in 2008. Otitis-prone children underwent adenoidectomy, myringotomy, and VT insertion, according to clinical practice guidelines [30]. The PCV7 was introduced in Greece in 2004; by 2006, it was included in the National Immunization Program. Almost all children had been vaccinated with the PCV7, as recommended by the National Immunization Program (2nd, 4th, 6th, and 12th month of age). The PCV13 was introduced near the end of 2010. In relation to pneumococcal vaccination, 266 patients were fully immunized with PCV7, 6 were partially immunized (two cases of 19A and one case with 19F), and one was not immunized. Study participants were rather older children, most of whom had already been immunized with PCV7 when PCV13 was introduced. Allergic rhinitis seems to play a role in COME and RAOM pathogenesis; therefore, patients with this condition were included in the study [31]. Patient history involving nasal membrane inflammation characterized by sneezing, rhinorrhea, nasal congestion, and itching in any combination, either perennial or seasonal, was the inclusion criteria. Children with a history of chronic otitis media, sensorineural hearing loss, craniofacial anomalies, immunosuppression, and cystic fibrosis were excluded from the study.

### 2.2. Clinical Specimens and Bacteriological Analyses

The external ear canal was disinfected with ear swabs soaked in alcohol prior to myringotomy. Radial-shaped myringotomy was performed under general anesthesia on the inferior anterior quadrant of the tympanic membrane using a standard aseptic technique.

### 2.3. Bacterial Cultures and Laboratory Testing

The swabs were transported in Amies medium to the microbiology department of our hospital as soon as possible and no longer than 2–4 h from the collection. Specimens were inoculated on Columbia agar containing 5% sheep blood and on chocolate agar. The inoculated plated media were incubated at 35 °C for 48 h in 5% to 10% CO_2_. Identification of microorganisms was performed by examining the morphology of the colonies, Gram stain, and other conventional methods such as sensitivity to optochin disk (5 mg) for *S. pneumoniae*, resistance to bacitracin disk (10 units), and V and X factor growth requirements for *H. influenzae*, positive oxidase test for *Moraxella catarrhalis*, positive catalase, and coagulase test for *S. aureus* and sensitivity to bacitracin disk (0.04 units) for *S. pyogenes*. Further identification of organisms was performed by Vitek GP (Biomerieux) for *S. pneumoniae*, *S. pyogenes*, and *S. aureus* and by Vitek NH (Biomerieux) for *H. influenzae* and *M. catarrhalis*. Susceptibility to antibiotics was tested by the standard disk diffusion method, according to CLSI guidelines.

### 2.4. Molecular Methods

Clinical samples such as middle ear aspirates were sent for molecular detection by means of multiplex polymerase chain reaction (mPCR) assays to the National Meningitis Reference Laboratory (NMRL). DNA isolation from clinical samples was carried out by the use of MagCore^®^ Genomic DNA Whole Blood Kit (MagCore HF 16 nucleic acid extraction system, RBC Bioscience, New Taipei City, Taiwan).

Molecular assays for the identification of the causative bacteria were performed by the NMRL, as described previously [32,33]. The first multiplex PCR assay [32] was used for the identification of *S. pneumoniae* and *H. influenzae* type b, and the second multiplex PCR assay [33] was used to identify *H. influenzae* non-type B, Streptococcus spp., *Staphylococcus aureus*, and *Pseudomonas aeruginosa* [32,33]. Among these bacteria, only those considered common AOM pathogens were included in the analysis, namely *S. pneumoniae*, *H. influenzae*, *H. influenzae* type b, *S. aureus*, and *Streptococcus pyogenes*. Another two mPCR assays were used to simultaneously detect nine common pneumococcal serotypes: (i) 4, 18C, serogroup 6, 23F, 19F, and (ii) 19A, 14, 3, 1, respectively, as previously described [34,35]. All PCV7 serotypes were examined with these assays with the exception of 9V, while among those included in the PCV13 serotypes, 5, 9V, and 7F were not included. *M. catarrhalis* strains were amplified by the use of primers based on the highly conserved regions of the eubacterial 16S rRNA as described elsewhere [36,37].

The multiplex PCR method, as applied in our protocol, holds several advantages that outcompete conventional microbiological methods of cultivation and substantially improve the detection rate of bacterial pathogens [38]. This molecular technique provides a precise characterization of the sample diversity by offering a more sensitive microbial qualitative profiling. Additionally, the sensitivity of the results is based on the fact that mPCR can detect (i) bacterial DNA in high rates of “culturally sterile” specimens [39] and (ii) the presence of multiple otopathogens in polymicrobial infections, including the biofilm-associated chronic otitis media [40,41].

### 2.5. In Vitro Biofilm Growth

All cultured bacteria were isolated and tested for their ability to form biofilms. The biofilms biomass metrics procedure was held at the Department of Biopathology and Clinical Microbiology of the “Aeginiteion” Hospital, Athens Medical School. Silicone squares of the same size (5 mm) were UV sterilized for 10 min and placed in Falcon tubes under aseptic conditions in a biological safety cabinet class IIB [42]. All bacterial strains isolated from the clinical samples by standard culture techniques were prepared in suspensions; *S. pneumoniae*, *Streptococcus* spp., *S. aureus*, and *M. catarrhalis*, were inoculated in 3 mL of Mueller-Hinton broth (MHB) (Scharlau™, Barcelona, Spain), while *H. influenzae* was inoculated in 3 mL of Brain-Heart infusion (BHI) broth (Scharlau™, Barcelona, Spain) supplemented with 5% lysed horse blood (Oxoid Ltd., Basingstoke, UK). Bacterial suspensions were incubated at 37 °C for 48 h at 150 rpm agitation to promote biofilm formation. After the incubation, the broth was removed with the aid of a vacuum pump device. The silicone discs were rinsed thrice with distilled water so that the non-adherent cells could be removed and left overnight to dry in a laminar flow hood.

### 2.6. Biofilm Dry Mass Metrics

For the biofilm biomass quantification, the silicone weighing methodology was followed as described elsewhere [43,44]. The silicone disks were cut into similar sizes (approximately 4 mm), weighed on a scale (25–30 mg), placed in falcon tubes, and left overnight to be sterilized under UV irradiation. Bacterial inoculation was achieved in the appropriate liquid medium (MHB and BHI as described above) incubated for 24 h at 37 °C. The liquid medium was poured off, and the tubes containing silicones were washed thrice with distilled water and left to dry in a laminar flow overnight. Then, tubes were weighed thrice at the accurate scales at two time points: (i) before the addition of the bacterial suspension and (ii) after the biofilms growth procedure; the difference in weight proved the growth of biofilms on the silicone surface. The average total biomass from two independent samples was calculated for each bacterial strain after subtracting the mass of a negative silicone control (no bacterial exposure).

### 2.7. Planktonic and Biofilm Antimicrobial Susceptibility Testing

Antimicrobial susceptibility testing of the planktonic bacteria was performed by the Kirby-Bauer standard disc diffusion method. Commercially-available pre-impregnated discs (Oxoid Ltd., UK), each containing a standard concentration of a particular antimicrobial, were used. Mueller-Hinton agar (Scharlau™, Barcelona, Spain) was used for all organisms except for *H. influenzae*, where Brain-Heart infusion agar supplemented with 5% lysed horse blood (Oxoid Ltd., UK) was applied. Colonies from the culture were uniformly inoculated on the agar surface, and plates were overnight incubated at 37 °C aerobically for all strains except for *H. influenzae*, which was incubated under a 5% CO_2_-enriched atmosphere. MIC determination was performed and interpreted according to the Clinical Laboratory Standard Institute (CLSI) guidelines [5,45,46].

Biofilm bacteria (sessile forms) were also studied for their ability to re-grow from the biofilm mass as planktonic forms in the presence of several routinely applied antimicrobials, including oxacillin, ampicillin, amoxicillin/clavulanic acid, clarithromycin, and cefotaxime following the serial microdilution method as described elsewhere [28]. In a flat-bottom 96-well microtiter plate, the selected antimicrobials were serially two-fold diluted (1:2) with the appropriate culture media (Brain-Heart infusion broth supplemented with lysed horse blood for *H. influenzae* and Mueller-Hinton broth for all the other bacterial strains) and one piece of biofilm-coated silicone was immersed in each well. The plate was incubated with gentle shaking at 37 °C for 48 h, and then the silicone discs were removed. The Minimal Inhibitory Concentration for Bacterial Regrowth (MICBR) was calculated as the first well without any visible precipitate on the bottom and absence of turbidity, indicating no bacterial regrowth. All microtiter assays were performed in duplicate. For the *H. influenzae* strains, the MICBR was calculated on the basis of positive subculture from each well because of the dark color of the medium and the difficulty of seeing the precipitate.

### 2.8. Statistical Analysis

To assess the relationship between pathogens and qualitative variables such as gender and clinical entity, we used Pearson’s chi-squared test of independence. The same test was used to assess the results of the antimicrobial susceptibility testing (comparison of the planktonic MIC with the respective biofilm MICBR) and the association of allergic rhinitis with recurrence and the need for re-operation. Moreover, Pearson’s chi-squared test was used to assess the association of clinical entity and myringotomy with recurrence and the need for re-operation. Paired *t*-test was used in order to assess the significance of the differences in the weights before and after the biofilm growth on the silicon disks for all infected samples together. The statistical analysis concerning the biofilm growth differences between COME and RAOM cases per pathogen was performed by using the Mann-Whitney U test, whereas for the comparison among pathogens, we used the Kruskal Wallis test followed by the appropriate post-hoc analysis. All statistical analyses were performed using the IBM SPSS Statistics 23.0, and *p* values < 0.05 were considered significant.

## 3. Results

A total of 273 patients (286 samples; ear effusion was collected from one or both ears according to the clinical presentation of the patient) hospitalized and operated on for complex OM were included in the study. There were 236 patients with COME (249 samples) and 37 patients with RAOM (37 samples). Of the 273 patients, there were 159 males and 114 females, while the median age was 58 months old (range: 6 months to 14 years).

### 3.1. Microbiological Findings

The pathogens identified in the middle ear fluid by culture and PCR in cases with COME and those with RAOM are shown in Table 1 and Table 2, respectively. The leading pathogen in otitis-prone cases was non-typeable *H. influenzae*. Among the COME cases, *H. influenzae* was identified in 19% (47/249) of the samples examined by standard culture methods and in 34.5% (86/249) of those examined with PCR. In addition, among the RAOM cases, *H. influenzae* was identified in 16% (6/37) of the samples examined by standard culture methods and in 40.5% (15/37) with the use of PCR.

*S. pneumoniae* was the second most common pathogen identified by PCR in 12.5% (31/249) of COME cases and in 24% (9/37) of RAOM cases, and by standard culture methods in 5% (12/249) and 5.5% (2/37) cases, respectively. Serotyping of the 40 *S. pneumoniae* strains from COME and RAOM showed that 35 were non-identifiable with the molecular method used; three were serotype 19A, one was serogroup 6, and one was serotype 19F. The two cases with 19A were RAOM cases, and the third was COME.

*M. catarrhalis* was the third most prevalent pathogen detected by PCR assays in both COME and RAOM cases, with a percentage of 6.4% (16/249) and 8.1% (3/37), respectively. Additionally, *M. catarrhalis* was identified by standard culture methods in both COME (5%, 12/249) and RAOM (2.7%, 1/37) cases.

*S. aureus* was identified in 14 (5.5%) samples from cases with COME and in 2 (5.5%) with RAOM. With regards to other bacteria isolated from middle ear fluid in culture (PCR not available), results are shown in Table 1 and Table 2.

Mixed infections with *H. influenzae* and *S. pneumoniae* were found in 3.5% (9/249) of COME samples and in 19% (7/37) of RAOM samples with the use of PCR. In a few COME cases, we identified the concurrent presence of *H. influenzae* and *Streptococcus* spp. (0.8%), *H. influenzae* and *S. aureus* (0.4%), and *S. pneumoniae* and *Streptococcus* spp. (0.4%). Moreover, in the 2.7% of the analyzed RAOM cases, *S. pneumoniae*-*M. catarrhalis* and *H. influenzae*-*Streptococcus* spp., co-infections were detected.

No statistically significant age-related microbiological findings were found. Regarding gender, *S. pneumoniae* was noted to be more common among female patients (*n* = 23/119, *p* = 0.028). No relation between the pathogen identified and the clinical entity (COME or RAOM) was found, apart from *S. pneumoniae*, which was significantly more common among patients with RAOM (*p* = 0.029) (Table 1 and Table 2).

### 3.2. Biofilm Production

The majority of bacterial species in both COME and RAOM cases seemed to produce biofilms. Of the total isolated strains, 83% (97/117) were positive for biofilm production on silicon discs, while of the common pathogens *H. influenzae*, *S. pneumoniae*, and *M. catarrhalis*, 94% (*n* = 64/68) were positive. Although the differences in the weights before and after the biofilm growth for all infected samples together were found to be particularly low (median = 0.009; IQR: 0.003–0.019), they were statistically significant (*p*-value = 0.001). The biofilm weight difference among COME and RAOM cases per pathogen as well as among the different pathogens regardless of the clinical entity are presented in Table 3. For all pathogens, there was no statistically significant difference in biofilm growth between COME and RAOM cases. However, for the total group of patients, the weight alterations in the *H. influenzae* and *S. pneumoniae* biofilms were significantly higher compared to those formed by *S. aureus* and *M. catarrhalis* (*p* = 0.002).

### 3.3. Antimicrobial Susceptibility Testing and Determination of Minimal Inhibitory Concentration for Bacterial Regrowth

The MIC of the planktonic bacterial forms was compared with the respective MICBR of the bacterial forms grown from the biofilms. MICBR values higher than the MIC CLSI breakpoints for antibiotic susceptibility suggest that these forms do not clinically respond to antimicrobial patient treatment.

The *H. influenzae* strains identified in COME samples and isolated by primary culture (planktonic) were found susceptible to a variety of antibiotics, such as ampicillin (100%), amoxicillin (100%), amoxicillin/clavulanic acid (100%), cefuroxime (78.7%), cefotaxime (89.4%), clarithromycin (89%) and ciprofloxacin (95.7%), according to MIC determination (Table 4). Moreover, regarding the RAOM samples, the planktonic *H. influenzae* strains were also found susceptible to ampicillin (83.3%), amoxicillin (83.3%), amoxicillin/clavulanic acid (100%), cefuroxime (100%), cefotaxime (100%), clarithromycin (100%), and ciprofloxacin (100%). In contrast, taking into consideration the MICBR data (Table 4), the *H. influenzae* strains grown from biofilms isolated from COME samples were highly resistant against the majority of the applied antibiotics. More specifically, these strains were found resistant to ampicillin (70.2%), amoxicillin (66%), amoxicillin/clavulanic acid (61.7%), and cefotaxime (55.3%) (with *p* < 0.0001 compared to their planktonic forms). Concerning the *H. influenzae* formatted biofilms from the RAOM samples, we observed high resistance to ampicillin (100%), amoxicillin (100%), and amoxicillin/clavulanic acid (83.3%) (*p* < 0.0001).

The planktonic *S. pneumoniae* strains isolated from COME samples were found susceptible to amoxicillin (100%), amoxicillin/clavulanic acid (100%), cefuroxime (83.3%), cefotaxime (83.3%), and levofloxacin (100%), while they were less susceptible to clarithromycin (75%). Similarly, the RAOM-derived planktonic *S. pneumoniae* strains were found to be highly susceptible against all the above-mentioned antibiotics (100%). The determination of the MICBR of the *S. pneumoniae* biofilm formation in both COME and RAOM cases revealed high resistance percentages to the antimicrobials tested. Among the COME samples, *S. pneumoniae* grown from biofilms were highly resistant to cefuroxime and cefotaxime (75%) (*p* < 0.0001 compared to their planktonic forms). Additionally, the grown strains showed moderate resistance to clarithromycin (58.3%), amoxicillin (50%), and amoxicillin/clavulanic acid (50%) (*p* < 0.001), while they remained susceptible to levofloxacin (83.3%). Regarding the RAOM samples, the *S. pneumoniae* strains grown from biofilms exhibited intermediate resistance to most antimicrobials (50%) (*p* < 0.001), except for levofloxacin, under the treatment of which the grown bacteria were totally susceptible (100%).

Similar to the aforementioned pathogens, the isolated from COME and RAOM cases planktonic *M. catarrhalis* strains were highly susceptible to amoxicillin/clavulanic acid, clarithromycin, and ciprofloxacin. Moreover, the *M. catarrhalis* strains grown from the biofilms of COME samples were highly resistant to clarithromycin (91.7%) (*p* < 0.0001), while they were less susceptible to amoxicillin/clavulanic acid (66.7%, 91.7%, respectively) compared to their planktonic forms.

Regarding the planktonic *S. aureus* strains isolated from the COME samples, we generally observed less susceptibility to the antibiotics compared to the other pathogens. Specifically, these strains were susceptible to Oxacillin (43.9%), clarithromycin (64.3%), and ciprofloxacin (63.6%), while the respective biofilm strains were totally resistant against the same treatments (*p* < 0.0001).

### 3.4. Allergic Rhinitis in Otitis-Prone Children

In 19.7% (40/203) of COME cases, the disease reoccurred, while 5.4% (11/203) of the patients were re-operated. Among the patients that COME reoccurred, 40% (16/40) had allergic rhinitis, and the respective percentage for the re-operated COME cases was 63.6% (7/11). Meanwhile, from the rAOM group, 46% (16/35) had another AOM episode, and 23% (8/35) were re-operated. Among the RAOM patients, a history of allergic rhinitis was noted in 43% (15/35), in 44% (7/16) of those whose AOM reoccurred, and in 25% (2/8) of those who re-operated. There seems to be a strong relevance of allergic rhinitis with recurrence as well as the need for re-operation for either entity (*p* < 0.001).

### 3.5. Follow-Up

A follow-up interview was conducted through telephone communication, approximately two to four years postoperatively (median 38 months, range 24–50 months). A small number of patients were not available for follow-up (48/286). Thus, a total of 238 patients were ultimately enrolled in the follow-up study concerning the recurrence of effusion and the need for a second or even a third operation (Table 5, Table 6 and Table 7). According to Table 7, the recurrence and the need for re-operation were higher for RAOM cases compared to those with COME regardless of the pathogen or whether VTs were inserted or not (*p* = 0.002 for both recurrence and re-operation). Regardless of the clinical entity, no significant difference was revealed comparing cases with and without ventilation tubes (VT) for both recurrence and re-operation (*p* = 0.196 and *p* = 0.286, respectively). Comparing myringotomy with and without VT in terms of recurrence, no significant difference was revealed for both COME and RAOM cases (*p* = 0.134 and *p* = 0.765, respectively). Similarly, comparing myringotomy with and without VT in terms of re-operation, no significant difference was revealed for both COME and RAOM cases (*p* = 0.331 and *p* = 0.999, respectively).

## 4. Discussion

In the present study, we prospectively investigated the presence of common AOM pathogens in the middle ear effusion of otitis-prone children in the post-PCV7 era. It was found that these pathogens are implicated with non-typeable *H. influenzae* being the predominant pathogen, whereas *S. pneumoniae* PCV7 serotypes were uncommon. Mixed bacterial infections, allergic rhinitis, and biofilms seem to be associated with otitis-prone cases.

The common AOM pathogens, including non-typeable *H. influenzae*, *S. pneumoniae*, and *M. catarrhalis* were identified in the middle ear of otitis-prone children. It was confirmed that PCR is a more sensitive method in identifying pathogens in the middle ear of complex OM cases compared to culture, as presented in Table 1 and previously shown [3,39,47,48,49]. It was also found with the use of PCR that polymicrobial infections are common, and they are difficult to diagnose with conventional microbiological culture methods [22,25,49]. In accordance with previous studies, it was shown that non-typeable *H. influenzae* is the leading pathogen identified in otitis-prone cases, while *S. pneumoniae* rates second [3,22,49,50,51,52]. Although *H. influenzae* was the predominant pathogen in both entities, *S. pneumoniae* seems to be more often associated with RAOM compared to COME.

Interestingly, there was a high percentage of *H. influenzae*–*S. pneumoniae* mixed infections in RAOM identified especially by PCR in agreement with other studies [20,29,52,53,54]. As it is reported, these two pathogens form a dual biofilm community that provides indirect pathogenicity [9,53,55,56,57]. This may be another reason for the difficulty in treatment, and the high recurrence rate, as polymicrobial infections, can have a profound impact on the course of the disease [58]. Our findings, coupled with the introduction of the conjugate pneumococcal vaccines, are in accordance with results from all over the world [20].

*S. pneumoniae* PCV7 serotypes were not identified. This most likely reflects the effect of immunization with PCV7 on the epidemiology in otitis-prone children [49]. Previous studies conclude the following: (i) PCV7 effectively prevents AOM (reduced visits and antimicrobial prescriptions for OM) [59,60], while it also reduces otitis-prone cases (reduced VT placement) [20,60,61,62]; (ii) serotype replacement was noted, especially by 19A which was reported to be highly resistant and implicated in complicated AOM [63,64]; (iii) no significant reduction in acute mastoiditis caused by *S. pneumoniae* [60]; (iv) significant reduction up to 97% was noted for invasive pneumococcal diseases in comparison to moderate reduction in OM visits (6–9%) [65]; (v) changes of a predominant pathogen for AOM between *S. pneumoniae* (pre-PCV era), *H. influenzae* and non-PCV7 pneumococcal serotypes (post-PCV era) [49], and (vi) early pneumococcal immunization is very important as there seems to be no reduction in AOM when PCV7 was administered in patients with RAOM [60]. Early pneumococcal OM (<6 months) causes middle ear damage that predisposes to otitis-prone conditions from non-vaccine serotypes and other otopathogens, mainly non-typeable *H. influenzae* [66]. On the contrary, a Finish study has shown that early all-cause AOM does not predispose to a higher risk of future AOM [67]. Especially for Greece, where PCV7 was introduced in the immunization system in 2006, the rate of pneumococcal disease decreased, and *S. pneumoniae* was replaced by *H. influenzae* in complex AOM cases with otorrhea [68].

Recent studies comparing the pre-PCV with PCV7 and PCV13 eras have shown a reduction in OM (overall 68%) after the PCV13, not only for *S. pneumoniae* but also for *H. influenzae*, *M. catarrhalis* and even culture-negative OM [66,69]. It should be emphasized that all-cause OM was not reduced until the post-PCV13 era depicting the importance of PCVs and, most importantly, early-life pneumococcal immunization [66]. Interestingly, an increase in nasopharyngeal carriage of non-vaccine serotypes was not followed by an increase in complex OM [66].

In our present study, serotype SP 19A was not found among the implicated ones. This is in accordance with other studies [29]. A previous report from our institution on complicated or refractory AOM during 2010–2011 showed that *S. pneumoniae* 19A was the predominant pathogen [63]. It is unlikely that this finding is related to the introduction of PCV13 because most cases were enrolled in the current study before or shortly after the introduction of PCV13. This finding suggests that different serotypes are implicated in each clinical entity as they differ in biofilm-forming ability and disease potential [9,29,69,70,71].

The microbiological procedures showed that isolated common AOM pathogens could form biofilms in vitro at a high rate (94%), as described elsewhere [9]. Other studies have shown biofilm existence in vivo, as well as intracellular persistence of pathogens in the host’s cells [54]. Thus, they may play an important role in otitis-prone pathogenicity and explain the ineffectiveness of the current antimicrobial strategy used. This may be due to (i) the inherent resistance of biofilms to antimicrobials attributed to the matrix material that bacteria are embedded in, (ii) the reduced metabolic activity [72], and (iii) the distinct biofilm phenotype they have adopted through *quorum sensing* [73], or lateral gene transfer in multiple colonization cases [70]. There are quorum signals that enable bacteria to be resistant to antibiotics that otherwise would be sensitive [56,58,74]. The amount of biofilm production may not depend on the pathogen or the clinical entity. It has been shown that *H. influenzae* strains from the nasopharynx of healthy children seem to produce more biofilm than *H. influenzae* isolated from AOM [71]. In addition, *H. influenzae*, along with *S. pneumoniae*, does not seem to produce more biofilm in vitro than *H. influenzae* alone [71]. On the other hand, in vivo, dual biofilm seems to be bigger in the chinchilla model AOM experiment [56]. The MICBR results of the current study are in accordance with published data showing that antibiotics are not an effective therapeutic solution in COME cases, as treatment failure may be anticipated [56,75,76]. A previous review pointed out that to cure one child with COME, eight children should be prescribed antimicrobials; thus, they should not be routinely used in COME [75]. However, we observed lower resistance levels in COME-derived biofilms upon treatment with cephalosporins and quinolones compared to penicillins, suggesting the former antibiotics as a better approach.

Biofilms in adenoids, according to the literature, seem to play a role in COME [77], though there is a conflict about whether they are strongly related to adenoidal microbiota [9,78] or not [10,47]. The nasopharyngeal carriage seems not to be affected dramatically in the post-PCV era as the *H. influenzae* was not affected, vaccine serotypes were replaced by other non-vaccine serotypes [79], and other pathogens such as *S. aureus* seem to rise [80]. Interestingly, the degree of bacterial colonization seems to be more important than the actual size of adenoids in the pathogenesis of COME [81].

The established therapeutic management of COME and RAOM with myringotomy and VT insertion does not always lead to a cure, and about 20–25% of the patients will require a second operation within two years [4,77]. In our study, regardless of the causative pathogen, patients who underwent myringotomy with or without the insertion of VT for COME were less often re-operated (Table 7). Meanwhile, the need for reoperation in RAOM cases is according to previous reports (Table 7). Interestingly, there was a higher rate of reoperation among *H. influenzae* RAOM over COME cases (Table 5 and Table 6). Myringotomy with or without VT insertion seems to work well in most of the cases independently of the causative pathogen; however, a few readmissions were noted in our study. Concrete data regarding readmission rates after myringotomy with or without VT insertion are not widely available in the current literature and are highly dependent on cofactors additional to the surgical procedure [79,80].

In addition, cases complicated with allergic rhinitis also seem to be prone to re-operation. Allergic rhinitis is among the key determinants of complex OM pathogenesis [81], as the accompanying swelling and obstruction of the Eustachian tubes lead to a prolonged middle-ear effusion [16,82,83].

The main limitations of our study lie firstly in the use of an in vitro method for the detection of biofilm production and the fact that we did not have a comparison group of strains causing acute otitis media or strains of the above organisms from healthy carriers to compare them. The standard gold method of confocal laser scanning microscopy was not available for biofilm study, so the silicone-weighing in vitro method was used instead. However, such methods for research purposes are encouraged so as to get an insight into the biofilm’s basic biology and tolerance, even though not every parameter of their in vivo existence can be addressed [84]. The number of RAOM cases was quite low because it is not easy to find middle ear fluid in such pathology, yet it is considered representative as these 37 cases were recruited in a major children’s hospital in Athens during the study period. Moreover, a limited number of *S. pneumoniae* serotypes were examined as PCV7, and the number of PCV13 additional serotypes was mainly targeted to assess the impact of PCV7 on otitis-prone cases. Finally, no nose samples were obtained from the COME and RAOM children with allergic rhinitis, and thus we could not verify the presence of the same bacterial species in the nose. This has to be addressed in the future to validate the strong relevance of allergic rhinitis with the recurrence of otitis media and the need for re-operation detected in the current study.

Despite the limitations, this is the first study investigating the prevalence of bacterial pathogens and biofilm formation in middle ear aspirates from children with COME and RAOM undergoing myringotomy in one of the largest Greek pediatric hospitals. Extensive electronic literature searches provided information on the current landscape of the microbiological profile research in the otitis-prone Greek children population. Queries were conducted to search the literature for the last 20 years (2012–2022) by using the following keywords in PubMed searches: “otitis media”, “effusion”, “biofilm”, and “Greece”. Articles without an abstract or papers with irrelevant content were excluded from the search process. The prevalence of otitis media in children has been widely investigated worldwide, but only a few studies have been performed in Greece. Since 2012, there have been 117 results for the queries “otitis media” AND “Greece”, and 34 results for “otitis media” AND “effusion” AND “Greece”. As for the keywords “recurrent otitis media” or “acute otitis media” AND “biofilm” AND “Greece” there are no results in PubMed, though only two for the queries “otitis media” AND “biofilm” AND “Greece”. The last two searches refer to literature reviews published in 2007 [85,86]. The gap in the literature and research in the field of biofilm-related polymicrobial infections in otitis-prone children is a major drawback in therapeutic approaches, even in the post-immunization era.

Methodological tools providing insights into the presence of polymicrobial infections, like in otitis-prone cases, will substantially support the continuous follow-up of microbiology worldwide. However, much remains to be learned about the mechanisms by which bacteria within biofilms resist both the host immune system and antimicrobials in order to make therapeutics more targeted and effective. Hopefully, PCV13 will further diminish in future otitis-prone cases, both pneumococcal and non-pneumococcal, as is already depicted [66].

## 5. Conclusions

This perspective study focused on the microbiology of otitis-prone children. *H. influenzae* still seems to be the leading pathogen implicated, while no *S. pneumoniae* PCV7 serotypes were detected. Mixed infections were common, and their ability to form in vitro biofilms might explain the difficulty in treatment. Interestingly, *S. pneumoniae* serotype 19A, which for the same period was detected in mastoiditis and other complicated acute otitis media cases in the same institute, was not detected in otitis-prone children. Thus, different *S. pneumoniae* serotypes might be implicated in different clinical entities. Myringotomy with or without ventilation tube insertion seems to work well in otitis-prone cases, especially in COME.

## Figures and Tables

**Table 1:** Pathogen identification in COME samples by culture and PCR.

COME Samples (*n* = 249)	Culture (+) and PCR (+)	PCR (+) and Culture (−)	Total PCR (+)	PCR (−) and Culture (+)	Culture (+), PCR N/A
*H. influenzae*	47 (19%)	39 (15.5%)	86 (34.5%)	0	
*S. pneumoniae*	12 (5%)	19 (7.5%)	31 (12.5%)	0	
*M. catarrhalis*	12 (5%)	4 (1.6%)	16 (6.4%)	N/A	
*S. aureus*	14 (5.5%)	0	14	0	
Other/Normal flora	N/A	N/A	N/A	N/A	53 (21%)
Mixed infections					
*H. influenzae* and *S. pneumoniae*	4 (1.6%)	5 (2%)	9 (3.5%)	0	
*H. influenzae* and *S.* spp.	2 (0.8%)	0	2 (0.8%)	0	
*H. influenzae* and *S. aureus*	1 (0.4%)	0	1 (0.4%)	0	
*S. pneumoniae* and *S.* spp.	1 (0.4%)	0	1 (0.4%)	0	

With respect to the pathogens detected by PCR, there was a complete concordance with culture. N/A: non-applicable, some bacteria were not determined by mPCR.

**Table 2:** Pathogen identification in RAOM samples by culture and PCR.

RAOM Samples (*n* = 37)	Culture (+) and PCR (+)	PCR(+) and Culture (−)	Total PCR (+)	PCR (−) and Culture (+)	Culture (+), PCR N/A
*H. influenzae*	6 (16%)	9 (24%)	15 (40.5%)	0	
*S. pneumoniae*	2 (5.5%)	7 (19%)	9 (24%)	0	
*M. catarrhalis*	1 (2.7%)	2 (5.3%)	3 (8.1%)	N/A	1 (2.7%)
*S. aureus*	2 (5.5%)	0	2 (5.5%)	0	
*Streptococcus* spp.	2 (5.5%)	2 (5.5%)	4 (11%)	0	
Other/Normal flora	N/A	N/A	N/A	N/A	8 (21.5%)
Mixed infections					
*S. pneumoniae* and *H. influenzae*	1 (2.7%)	6 (16%)	7 (19%)	0	
*S. pneumoniae* and *M. catarrhalis*	0	1 (2.7%)	1 (2.7%)	0	
*H. influenzae* and *S.* spp.	1 (2.7%)	0	1 (2.7%)	0	

With respect to the pathogens by PCR, there was a complete concordance with culture. N/A: non-applicable, some bacteria were not determined by mPCR.

**Table 3:** Biofilm weight differences among COME and RAOM cases.

Pathogen	Total	COME	RAOM	*p* Value
Median (IQR)				
*Haemophilus influenzae*	0.011 (0.008–0.020)	0.013 (0.008–0.021)	0.010 (0.005–0.015)	0.354 *
*Streptococcus pneumoniae*	0.013 (0.002–0.025)	0.010 (0.002–0.029)	0.008 **	
*Staphylococcus aureus*	0.005 (0.003–0.008)	0.005 (0.003–0.008)	0.004 (0.001–0.007)	0.533 *
*M.catarrhalis*	0.005 (0.003–0.020)	0.005 (0.003–0.018)	0.019 **	
*p* Value	0.002 ^#^	0.244 ^#^	-	

ΙQR: interquartile range; * *p* values were calculated with the non-parametric Mann-Whitney U test; # *p* values were calculated with the non-parametric Kruskal Wallis test, followed by Dunn’s test for multiple comparisons; ** IQR not applicable due to the limited number of samples.

**Table 4:** Planktonic MIC and Biofilm MICBR of the antimicrobials of all isolates for COME and RAOM.

*H. influenzae*					
Antimicrobial	COME		RAOM		Breakpoints (susceptible)
	μg/mL (% S or R)		μg/mL (% S or R)		
	Planktonic MIC	Biofilm MICBR	Planktonic MIC	Biofilm MICBR	
Ampicillin	≤1 (100 S)	>4 (70.2 R)	≤1 (83.3 S)	>4 (100 R)	≤1
Amoxicillin	≤1 (100 S)	>4 (66.0 R)	≤1 (83.3 S)	>4 (100 R)	≤1
Amoxicillin/Clavulanic acid	≤2 (100 S)	≥8 (61.7 R)	≤2 (100 S)	≥8 (83.3 R)	≤4/2
Cefuroxime	<4 (78.7 S)	<4 (66.0 S)	<4 (100 S)	<4 (66.7 S)	≤4
Cefotaxime	<2 (89.4 S)	≥8 (55.3 R)	≤2 (100 S)	≤2 (83.3 S)	≤2
Clarithromycin	<8 (89.4 S)	<8 (68.8 S)	<8 (100 S)	<8 (100 S)	≤8
Ciprofloxacin	≤1 (95.7 S)	≤1 (51.1 S)	≤1 (100 S)	≤1 (100 S)	≤1
*S. pneumoniae*					
Antimicrobial	COME		RAOM		Breakpoints (susceptible)
	μg/mL (% S or R)		μg/mL (% S or R)		
	Planktonic MIC	Biofilm MICBR	Planktonic MIC	Biofilm MICBR	
Amoxicillin	≤2 (100 S)	≤2 (50 S)	≤2 (100 S)	≤2 (50 S)	≤2
Amoxicillin/Clavulanic acid	<2 (100 S)	≤1 (50 S)	≤2 (100 S)	≤2 (50 S)	≤2/1
Cefuroxime	≤0.5 (83.3 S)	>4 (75 R)	≤0.5 (100 S)	≤0.5 (50 R)	≤0.5
Cefotaxime	≤0.5 (83.3 S)	>4 (75 R)	≤0.5 (100 S)	≤0.5 (50 R)	≤0.5
Clarithromycin	≤0.25 (75 S)	>2 (58.3 R)	≤0.25 (100 S)	≤0.25 (50 R)	≤0.25
Levofloxacin	≤2 (100 S)	≤2 (83.3 S)	≤2 (100 S)	≤2 (100 S)	≤2
*M. catarrhalis*					
Antimicrobial	COME		RAOM		Breakpoints (susceptible)
	μg/mL (% S or R)		μg/mL (% S or R)		
	Planktonic MIC	Biofilm MICBR	Planktonic MIC	Biofilm MICBR	
Amoxicillin/Clavulanic acid	≤2 (83.3 S)	≤2 (66.7 S)	≤2 (100 S)	≤2 (100 S)	≤4/2
Clarithromycin	≤1 (91.7 S)	>4 (91.7 R)	≤1 (100 S)	≤1 (100 S)	≤1
Ciprofloxacin	≤1 (100 S)	≤1 (91.7 S)	≤1 (100 S)	≤1 (100 S)	≤1
*S. aureus*					
Antimicrobial	COME		RAOM		Breakpoints (susceptible)
	μg/mL (% S or R)		μg/mL (% S or R)		
	Planktonic MIC	Biofilm MICBR	Planktonic MIC	Biofilm MICBR	
Oxacillin	≥4 (57.1 R)	≥4 (100 R)	≥4 (100 R)	≥4 (100 R)	≤2
Clarithromycin	≤2 (64.3 S)	≥8 (100 R)	≤2 (100 S)	≥8 (100 R)	≤2
Ciprofloxacin	≤1 (63.6 S)	≥4 (100 R)	≤1 (100 S)	≥4 (100 R)	≤1

MIC: minimum inhibitory concentration; MICBR: minimum inhibitory concentration for bacterial regrowth; S: susceptibility; R: resistance.

**Table 5:** Follow-up of COME patients.

Pathogen	PCR (+)	Follow-Up	Transient OME	Re-Operation
*H. influenzae*	86	37	6 (16%)	2 (5.5%)
*S. pneumoniae*	31	11	2 (18%)	0
*M. catarrhalis*	16	12	2 (16.5%)	1 (8.5%)
*S. aureus*	14	10	2(20%)	1 (10%)
Other/Negative		133	26(19.5%)	7 (5%)
**Total**		203	38(18.7%)	11(5.4%)

**Table 6:** Follow-up of RAOM patients.

Pathogen	PCR (+)	Follow-Up	Transient OME-RAOM	Re-Operation
*H. influenzae*	15	5	3 (60%)	1 (20%)
*S. pneumoniae*	9	2	0	0
*M. catarrhalis*	1	1	0	0
*S. aureus*	2	2	1 (50%)	1 (50%)
Other/Negative		25	12 (48%)	6 (24%)
**Total**		35	16 (46%)	8 (22.9%)

**Table 7:** Recurrence and re-operation cases of COME and RAOM according to initial surgical procedure.

	Recurrence	Re-Operation
COME (*n* = 203)		
Myringotomy without VT (*n* = 74)	10 (13.5%)	2 (2.5%)
Myringotomy with VT (*n* = 129)	30 (23%)	9 (7%)
RAOM (*n* = 35)		
Myringotomy without VT (*n* = 9)	5 (55%)	2 (22%)
Myringotomy with VT (*n* = 26)	11 (42%)	6 (23%)
*p* Value *	0.002	0.002

VT: ventilation tubes; * *p* values were calculated with Pearson’s chi-squared test.

## Data Availability

All relevant data are within the manuscript.
